# A tissue engineering strategy for the treatment of avascular necrosis of the femoral head

**DOI:** 10.1016/j.surge.2013.02.008

**Published:** 2013-12

**Authors:** A. Aarvold, J.O. Smith, E.R. Tayton, A.M.H. Jones, J.I. Dawson, S. Lanham, A. Briscoe, D.G. Dunlop, R.O.C. Oreffo

**Affiliations:** Bone and Joint Research Group, Centre for Human Development, Stem Cells and Regeneration, University of Southampton, Institute of Developmental Sciences, Tremona Road, Southampton SO16 6YD, UK

**Keywords:** Femoral head, Avascular necrosis, Skeletal stem cells, Impaction bone grafting, Translation

## Abstract

**Background & purpose:**

Skeletal stem cells (SSCs) and impaction bone grafting (IBG) can be combined to produce a mechanically stable living bone composite. This novel strategy has been translated to the treatment of avascular necrosis of the femoral head. Surgical technique, clinical follow-up and retrieval analysis data of this translational case series is presented.

**Methods:**

SSCs and milled allograft were impacted into necrotic bone in five femoral heads of four patients. Cell viability was confirmed by parallel *in vitro* culture of the cell-graft constructs. Patient follow-up was by serial clinical and radiological examination. Tissue engineered bone was retrieved from two retrieved femoral heads and was analysed by histology, microcomputed tomography (μCT) and mechanical testing.

**Results:**

Three patients remain asymptomatic at 22- to 44-month follow-up. One patient (both hips) required total hip replacement due to widespread residual necrosis. Retrieved tissue engineered bone demonstrated a mature trabecular micro-architecture histologically and on μCT. Bone density and axial compression strength were comparable to trabecular bone.

**Conclusions:**

Clinical follow-up shows this to be an effective new treatment for focal early stage avascular necrosis of the femoral head. Unique retrieval analysis of clinically translated tissue engineered bone has demonstrated regeneration of tissue that is both structurally and functionally analogous to normal trabecular bone.

## Introduction

AVN of the femoral head is a disease that usually affects young adults, progressing to bone collapse and osteoarthritis in over 80% of untreated patients.^1^ Progression occurs even in 59% of asymptomatic patients.^2^ Most cases are idiopathic but it is commonly attributed to steroid therapy, chemotherapy, alcohol or sickle cell disease, whilst the chance of developing AVN after traumatic hip dislocation may be as high as 40%.^3^ Position and distribution of the necrotic bone in the femoral head have a bearing on prognosis^2,4^ however the most important predictor remains progression from an intact bony architecture (Ficat & Arlet Stage I or II) to loss of normal bone structure and involvement of articular cartilage (Ficat & Arlet Stage III or IV).^5^

Untreated cases therefore, even in the early and asymptomatic stages, have a high probability of requiring a total hip replacement (THR). Joint preserving therapies are therefore advocated by most authorities to prevent the progression to collapse. These include core decompression,^1^ electrical stimulation,^6^ tantalum trabecular metal rods,^7^ vascularised fibular grafts,^8^ fibular or tibial strut grafts,^9^ concentrated autologous bone marrow^10^ or hydroxyapatite rods coated with skeletal stem cells.^11^

Skeletal stem cells (SSCs) have been used for the treatment of avascular necrosis of the femoral head,^12^ though this treatment does not provide any structural support for the overlying cartilage. Impaction bone grafting (IBG) can provide mechanical support and has been used as a void-filler in revision hip surgery for over forty years^13^ and in the treatment of AVN.^14^ Whilst some remodelling has been demonstrated on histological specimens from IBG in acetabula and femora, areas of non-incorporated graft with associated necrosis, fibrocartilage and fibrosis remain in all specimens.^15–17^

SSCs can be combined with IBG to improve both the mechanical and biological characteristics of the graft.^18^ This novel technique has been translated from the laboratory to the clinic for the treatment of early stage AVN. We report our case series from this novel tissue engineering strategy. Two femoral heads, both in the same patient, have collapsed requiring THR. This has however provided the opportunity for retrieval of the human tissue engineered bone and for unique *ex vivo* analysis.

## Materials and methods

### Surgical technique

Milled allograft was prepared from fresh frozen femoral heads according to standard clinical practice. The patient was positioned laterally on the operating table and bone marrow was aspirated from the posterior iliac crest, rotating and re-angling the needle regularly to minimise contamination with venous blood. The marrow was concentrated in theatre by calibrated centrifugation (Marrowstim, Biomet, Swindon, UK) to isolate the nucleated cell fraction. This provided a concentrated pool of pleuripotent SSCs,^18^ which was mixed with the prepared allograft. The patient was then placed supine on the operating table to allow fluoroscopic guidance of instrumentation up the femoral neck into the necrotic area of the femoral head. Through a 2 cm incision in the lateral thigh, a channel was drilled over a guidewire into the sub-chondral bone and the necrotic bone was removed by curettage. Cell-seeded milled allograft was impacted retrograde into the channel using a 12 mm diameter Xchange tube saw (Stryker, Newbury, UK). Samples of allograft/SSC mix were retained for parallel *in vitro* analysis of cell viability. Patients maintained protected weight bearing for 6 weeks post-operatively and follow-up was by serial radiological and clinical examination.

### Patient cohort

Four patients, all with bilateral AVN, were treated at our institution using this tissue engineering strategy. Three of the patients presented with advanced disease in one hip, requiring contra-lateral THR (Table 1).

### Parallel *in vitro* assessment

The retained samples were cultured in basal media for 14, 28 and 42 days, with twice weekly media changes, prior to staining with CellTracker Green and ethidium homodimer (CTG-EH). Microscope images were recorded using Carl Zeiss Axiovision software Ver 3.0 via an AxioCam HR digital camera on an Axiovert 200 inverted microscope (Carl Zeiss Ltd, Welwyn Garden City, UK) under fluorescent light.

### Retrieval of specimens

In Patient 4, both femoral heads progressed to collapse requiring bilateral THR, on the right after 13 months and on the left after 19 months. The femoral heads were retrieved, with patient consent and prior ethical approval (LREC194/99/1), photographed and fixed in 4% paraformaldehyde (PFA) prior to further analysis.

### Microcomputed tomography (μCT)

The retrieved femoral heads were scanned using an Xtek Benchtop 160Xi scanner (Xtek Systems Ltd, Tring, UK) equipped with a Hamamatsu C7943 X-ray flat panel sensor (Hamamatsu Photonics, Welwyn Garden City, UK). Scan resolution was up to 31-μm, at 150 kV, 60 μA using a molybdenum target with an exposure time of 534 ms and 4× digital gain. Reconstructed volume images were analysed using VGStudio Max 1.2.1 software (Volume Graphics GmbH, Heidelberg, Germany).

### Histology

**Fig. 1:**
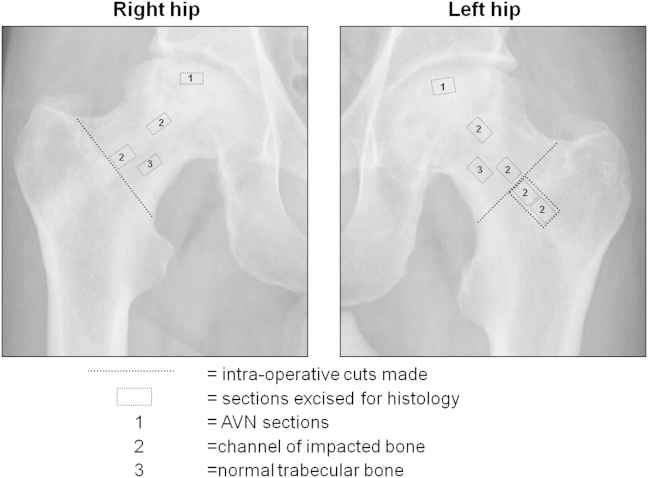
Schematic identifying the sections processed for histology.

For histological analysis of normal trabecular and impacted bone, representative sections were excised from the femoral neck, avoiding areas of collapse (Fig. 1, Sections ‘2’ and ‘3’ respectively). Sections of the collapsed necrotic tissue, were excised and processed in an identical fashion (Fig. 1, Section ‘1’). Cortical bone was not included. Specimens were decalcificied in 0.1 M TRIS/5% EDTA solution at pH 7.3 and decalcification verified by Faxitron MX-20 micro X-ray (Faxitron X-ray, Wheeling, IL, USA). After wax embedding, mounting, processing through graded ethanols and sectioning to 7 μm thickness, slides were stained with Alcian Blue/Sirius Red (A/S) and tartrate-resistant acid phosphatase (TRAP). Microscope images were recorded under white and polarised light on an Axiovert 200 inverted microscope (Carl Zeiss Ltd, Welwyn Garden City, UK).

### Mechanical testing

Sections of trabecular, cortical and impacted bone were excised from each specimen for comparison of mechanical strength. As described above, these sections were taken from the femoral neck region to avoid incorporating residual pathology. There was sufficient tissue to harvest two representative sections of each tissue type from each femoral head, providing four samples of each. Cuboidal bone sections (6.0 × 7.0 × 4.0 mm) were prepared using a low-speed diamond-tipped wafering blade (Buehler, Coventry, UK), calibrated to 0.1 mm accuracy using electronic callipers (Jade Products, Rugby, UK) and tested to failure by compression with a hydraulic actuator (Instron Ltd, High Wycombe, UK). A compressive force was applied at a constant rate of 4.8 mm over 60 s (0.08 mm/s), from which stress–strain graphs were plotted and mechanical strength calculated. In order to represent the forces sustained *in vivo*, the direction of force applied was always in the orientation of the trabecular stress lines of the femoral neck. Statistical significance was examined by One-way ANOVA.

## Results

### Clinical follow-up

Three patients remain asymptomatic at 22- to 44-month follow-up, with no radiological evidence of collapse. One patient progressed to bilateral collapse requiring THR. This has afforded the unique opportunity to retrieve tissue engineered bone from a human case for *ex vivo* analysis.

### Reasons for collapse of femoral heads of Patient 4

At diagnosis, the necrotic area in the right hip was widespread across the sub-chondral bone. MRI analysis confirmed a high Mitchell score^4^ suggestive of more widespread necrosis than was initially suspected, and predictive of increased technical difficulty in effective curettage of the whole area. Such disease may therefore not have been conducive to treatment by such a percutaneous technique. For the left hip, despite maintenance of femoral head architecture on pre-operative radiographs, high signal could be identified on MRI. Diagnosis on CT scan of a cyst confirmed degenerative change that may be unsalvageable. Collapse in both femoral heads occurred away from and lateral to the impacted channel of bone, most likely due to residual necrotic bone.

### Parallel *in vitro* assessment

**Fig. 2:**
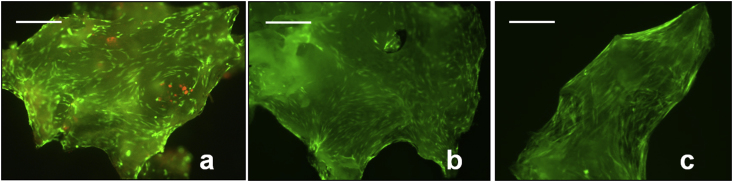
CTG-EH stain demonstrating enduring cell viability and proliferation on *in vitro* cell-seeded milled allograft. a: 2 weeks culture. b: 4 weeks culture. c: 6 weeks culture, with bridging of SSCs cross the construct. Scale bars = 200 μm.

*In vitro* culture of SSCs on allograft demonstrated enduring cell viability and proliferation of the impacted cells, evidenced by CTG-EH stain (Fig. 2a–c).

### Macroscopic analysis of retrieved femoral heads

**Fig. 3:**
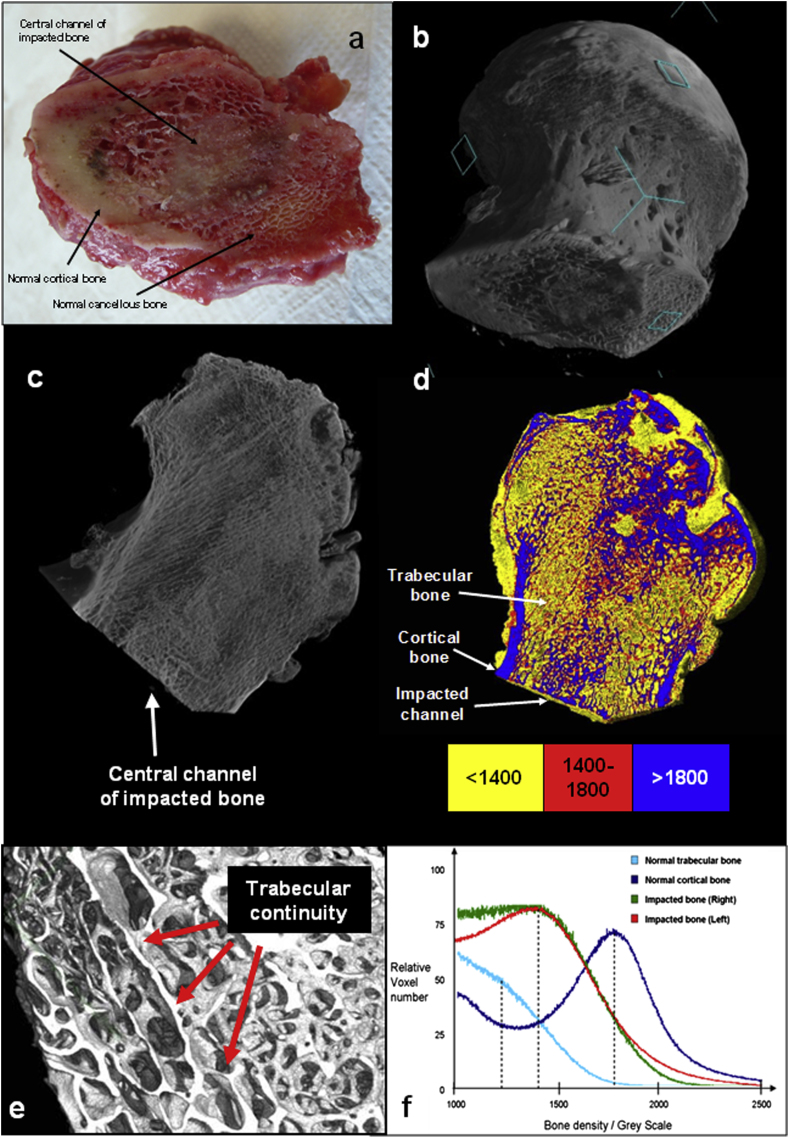
a: Photograph of the base of right femoral head of Patient 4 following surgical neck cut, showing a clearly demarcated dense central channel of remodelled impacted bone. b: 3D reconstructed μCT image at 70-μ resolution of retrieved right femoral head. c: Coronal section showing a dense channel of impacted bone up to the necrotic collapsed area. d: False-colour density analysis within right femoral head; Yellow <1400 Grey Scale units; Red = 1400–1800 units; Blue >1800 units. e: 3D reconstructed μCT image at 31-μ resolution of the central channel of impacted bone from the left femoral head. Continuous trabecular architecture is evident throughout the channel. f: Graph of density analysis, corresponding to Fig. 5, with peaks of trabecular, impacted and cortical bone seen at 1200, 1400 and 1800 units respectively, indicated by dashed lines.

Review of the cut surface of each femoral neck identified a demarcated dense central channel of bone, which continued through the femoral head and up to the collapsed sub-chondral bone (Fig. 3a).

### Microcomputed tomography (μCT)

Analysis by μCT revealed a dense central channel with a mature uniform trabecular structure visualised in cross-section along its length (Fig. 3b and c). With the exception of collapsed necrotic sub-chondral tissue, the trabecular micro-architecture was maintained, and observed to be in continuity throughout the impacted channel of bone (Fig. 3e).

The apparent bone density for trabecular, cortical and impacted bone was determined by Grey Scale on μCT images. As expected, bone densities in the right and left femoral heads were comparable to each other. Cortical bone was denser than trabecular bone, with a Grey Scale density of 1800 *versus* 1200 units. The density of the right and left impacted channels of bone were also comparable to each other, and greater than trabecular bone, with a Grey Scale density of 1400 units, illustrated by false-colour coding (Fig. 3d) and graphically (Fig. 3f).

### Histology

**Fig. 4:**
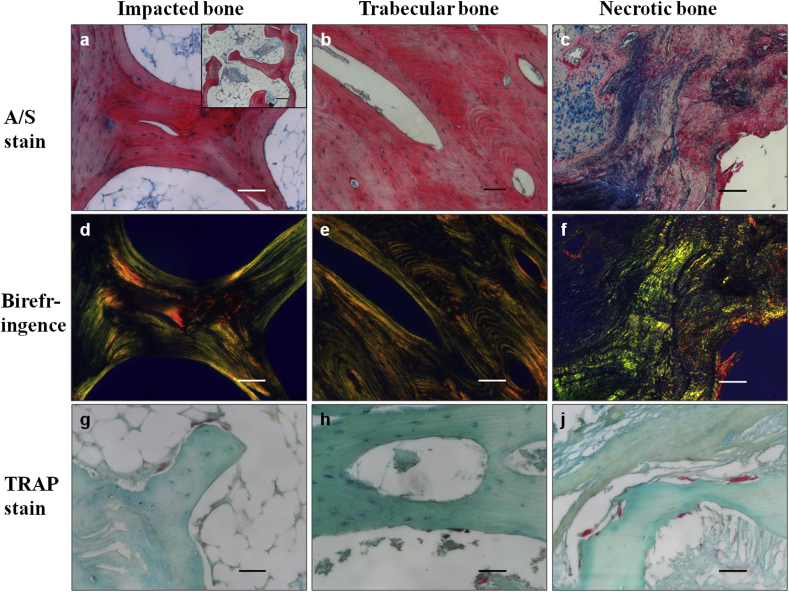
Histology; A/S stain of impacted bone revealed a trabecular structure (a, inset), with lamellae and cells within lacunae (a), comparable to trabecular bone (b); Parallel birefringence confirmed a mature ordered lamellar structure of both impacted and trabecular bone (d,e); The necrotic tissue demonstrated a disordered mixture of cartilage, fibrous tissue and bone (c), with negligible evidence of organisation when viewed under polarised light (f); TRAP staining demonstrated normal osteoclasts and Howship's lacunae in impacted bone (g) and trabecular bone (h) however a high number of osteoclasts could be observed in the necrotic bone (j). Scale bars: a–e = 100 μm; a inset, c,f = 200 μm; g,h,j = 50 μm.

A/S staining of tissue from the impacted channels of retrieved femoral bone showed histology consistent with normal trabecular bone. Osteocytes within lacunae were visible, surrounded by a lamellar structure (Fig. 4a) which was confirmed as mature organised bone under polarised light (Fig. 4d). The micro-architecture of the impacted bone was observed to be histologically identical to the patient's normal trabecular bone (Fig. 4b and e). Low power images of impacted bone confirmed the presence of a trabecular structure incorporating marrow (Fig. 4a, inset), though macroscopically and on μCT trabeculae were more densely arranged (Fig. 3a–d). TRAP stain revealed osteoclasts and Howship's lacunae on the surfaces of impacted bone (Fig. 4g) and normal trabecular bone (Fig. 4h). This microscopic arrangement was grossly different from tissue in the necrotic area which showed a high concentration of osteoclasts, eroded bone (Fig. 4j), and a disordered mixture of cartilage, fibrous tissue and bone (Fig. 4c). There was negligible evidence of organisation on birefringence (Fig. 4f).

### Mechanical testing

**Fig. 5:**
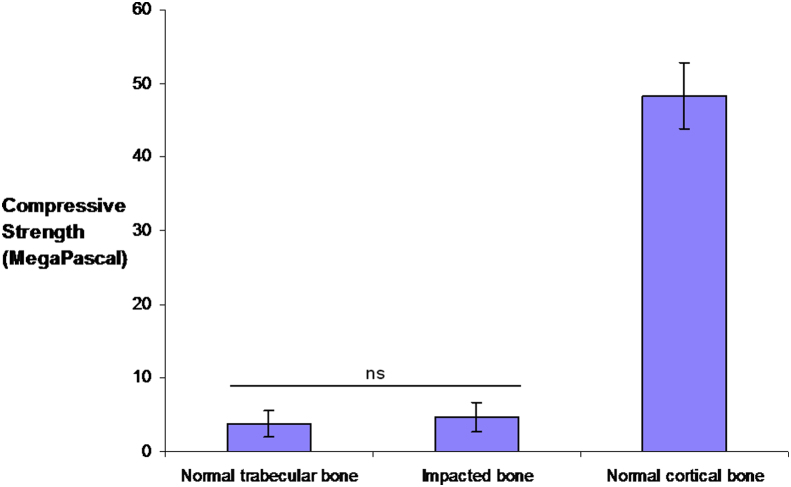
Bar chart demonstrating compressive strengths (MPa) of trabecular, impacted and cortical bone of both hips from Patient 4 (*n* = 4). One-way ANOVA demonstrated a statistical difference between trabecular and cortical bone (*p* < 0.0001), but no statistical difference between trabecular and impacted bone. Error bars denote Standard Error of Mean, ‘ns’ = no statistical significance.

Comparable values in compressive strength of the cortical, trabecular and impacted bone were found between right and left femoral heads (Fig. 5). One-way ANOVA test demonstrated statistical difference between the compressive strength of trabecular and cortical bone, which was confirmed on Bonferroni Multiple Analysis (*n* = 4, *p* < 0.05). Critically, there was no statistical difference in the strengths of trabecular and impacted bone (*p* > 0.05).

## Discussion

This paper describes a technique which appears to be effective for the treatment of AVN of the femoral head and, critically, is simple, quick and inexpensive. It supplements each of the current therapies of core decompression,^1^ SSCs^12^ and IBG^14^ by providing both a biological stimulus for osteogenesis as well as mechanical support for the sub-chondral bone. Three patients remain asymptomatic at up to 44-month follow-up. Furthermore, the *ex vivo* analysis of the two collapsed femoral heads has demonstrated the regeneration of structurally and functionally normal bone.

The continuous trabecular architecture throughout the impacted channel of bone observed by μCT (Fig. 3b, c and e) provides evidence of remodelling of the milled allograft, to create a mature living bone. With the exception of retained pathology in the sub-chondral bone, critically, no necrotic/fibrotic tissue was observed within the impacted channel, indicating all impacted fragments had been integrated, resorbed or remodelled.

Histological analysis confirmed the presence of a mature trabecular bone structure (Fig. 4a, inset). Osteoclasts and Howship's lacunae were indicative of normal remodelling (Fig. 4c) and the lamellar micro-architecture of the impacted bone, with osteocytes in lacunae, was observed to be histologically identical to normal trabecular bone (Fig. 4a and b). These results indicate SSCs together with the impacted milled allograft resulted in the formation of a bone composite that underwent remodelling. This must be contrasted with previous histological analysis performed on retrieval specimens of IBG without supplementation with SSCs, whereby islands of non-incorporated necrotic graft remained in all specimens.^15–17^

Compression testing demonstrated that the regenerated bone was of comparable strength to normal ipsilateral trabecular bone. An aggregate of milled allograft, as originally impacted into the patient, would crumble under minimal loading, yet despite no lateral constraints *ex vivo* the test segments of impacted bone maintained their shape indicating a significant increase in inter-particulate cohesion of the milled fragments. μCT scans confirmed that the fragments were conjoined, which together with the compression testing accounted for the structural integrity. μCT scans confirmed that the fragments were conjoined, which together with the compression testing accounted for the structural integrity.

## Conclusion

While clinical translation of tissue engineering strategies remains rare, this novel case series and *ex vivo* analysis has confirmed the potential of SSC/IBG constructs for the treatment of early stage femoral head AVN. Further clinical trials are necessary, including comparison to concurrent therapies, though referral can be made to extensive *in vitro* and *in vivo* work regarding SSC augmentation of bone regeneration on IBG.^18–20^ This technique may offer potential for treatment of the broader spectrum of bone defects.

## Sources of financial support

Surgical costs were funded by the National Health Service and laboratory costs were funded by grants from the Biotechnology and Biological Sciences Research Council (BBSRC) and the Technology Strategy Board (TSB).

## Figures and Tables

**Table 1:** Patient details.

Patient	Age	Attributed cause	Right hip stage^a^	Right hip treatment	Left hip stage^a^	Left hip treatment
1	42	Systemic steroids for sub-arachnoid haemorrhage	IV	THR	II	SSC/IBG
2	40	Alcohol	III	THR	II	SSC/IBG
3	31	Idiopathic	III	THR	II	SSC/IBG
4	32	Systemic steroids for testicular carcinoma	II	SSC/IBG	II	SSC/IBG

a = Ficat & Arlet Classification is used throughout this study unless otherwise stated.
